# Loss of symbiotic and increase of virulent bacteria through microbial networks in Lynch syndrome colon carcinogenesis

**DOI:** 10.3389/fonc.2023.1313735

**Published:** 2024-02-05

**Authors:** Mohammad Sadeghi, Denis Mestivier, Etienne Carbonnelle, Robert Benamouzig, Khashayarsha Khazaie, Iradj Sobhani

**Affiliations:** ^1^ EA7375 –EC2M3: Early detection of Colonic Cancer by using Microbial & Molecular Markers Paris East Créteil University (UPEC), Créteil, France; ^2^ Bacteriology, Virology, Hygiene Laboratory, Assistance Publique–Hôpitaux de Paris (APHP), Avicenne Hospital, Bobigny, France; ^3^ Department of Gastroenterology, Assistance Publique–Hôpitaux de Paris (APHP), Avicenne Hospital, Bobigny, France; ^4^ Department of Immunology, Mayo Clinic, Scottsdale, AZ, United States; ^5^ Department of Gastroenterology, Assistance Publique–Hôpitaux de Paris (APHP), Henri Mondor Hospital, Créteil, France

**Keywords:** gut microbiota, cancer, microbial network inference, Lynch syndrome, colorectal cancer

## Abstract

**Purpose:**

Through a pilot study, we performed whole gut metagenomic analysis in 17 Lynch syndrome (LS) families, including colorectal cancer (CRC) patients and their healthy first-degree relatives. In a second asymptomatic LS cohort (n=150) undergoing colonoscopy-screening program, individuals with early precancerous lesions were compared to those with a normal colonoscopy. Since bacteria are organized into different networks within the microbiota, we compared related network structures in patients and controls.

**Experimental design:**

Fecal prokaryote DNA was extracted prior to colonoscopy for whole metagenome (n=34, pilot study) or 16s rRNA sequencing (validation study). We characterized bacteria taxonomy using Diamond/MEGAN6 and DADA2 pipelines and performed differential abundances using Shaman website. We constructed networks using SparCC inference tools and validated the construction’s accuracy by performing qPCR on selected bacteria.

**Results:**

Significant differences in bacterial communities in LS-CRC patients were identified, with an enrichment of virulent bacteria and a depletion of symbionts compared to their first-degree relatives. Bacteria taxa in LS asymptomatic individuals with colonic precancerous lesions (n=79) were significantly different compared to healthy individuals (n=71). The main bacterial network structures, constructed based on bacteria-bacteria correlations in CRC (pilot study) and in asymptomatic precancerous patients (validation-study), showed a different pattern than in controls. It was characterized by virulent/symbiotic co-exclusion in both studies and illustrated (validation study) by a higher *Escherichia*/*Bifidobacterium* ratio, as assessed by qPCR.

**Conclusion:**

Enhanced fecal virulent/symbiotic bacteria ratios influence bacterial network structures. As an early event in colon carcinogenesis, these ratios can be used to identify asymptomatic LS individual with a higher risk of CRC.

## Translational relevance

Individuals with hereditary Lynch Syndrome (LS), have a significantly elevated risk of developing CRC and require inclusion in colonoscopy-based screening programs. However, current risk assessments do not consider environmental factors, and there is a lack of a reliable screening test to identify those in need of early, consecutive colonoscopies. We characterized the bacterial composition of the colonic microbiota, which is greatly influenced by environmental factors and found notable differences in microbial compositions. Patients diagnosed with cancer exhibited dysbiosis in comparison to their first-degree relatives. In a second LS cohort, individuals with early-stage colonic neoplasia also exhibited distinct bacterial compositions in contrast to those with normal colonoscopy confirming the existence of a gut bacteria network closely associated with cancer and precancerous lesions. Now, we wish developing innovative microbiota-related strategies for the early detection of colon neoplasia to improve existing screening programs, offering personalized medical care in LS, and characterizing novel checkpoints for therapy.

## Introduction

Colorectal cancer (CRC) is among the three most common cancers, with more than 1.2 million new cases and about 600,000 deaths per year worldwide (1). The majority of CRCs cases are classified as sporadic, meaning they arise from somatic gene alterations such as mutations and methylation changes. This observation suggests that environmental factors play a pivotal role in the development of CRC carcinogenesis (2). The gut bacterial community, which comprises a higher number of cells and functional genes compared to the host, plays a crucial role in maintaining host homeostasis. Changes in microbial communities, known as dysbiosis, are associated with various diseases, including CRC. Furthermore, dysbiosis is now recognized as an indicator of the environment’s influence on the development of emerging chronic diseases (3).

CRC occurs because of a series of oncogenic events that lead to hyperproliferation of colonic epithelial cells. This process involves the development of aberrant crypt foci (ACF) and the sustained transition from adenoma to *in situ* carcinoma, tumor invasion, tumor vascularization, and spread metastases (4). Only less than 5% of CRCs are due to hereditary gene alterations (5). These include mostly Lynch syndrome (LS) individuals who harbor germline mutations in the DNA mismatch repair (MMR) gene system (5, 6). However, even in these cases, the role of the environment may be critical in the development of cancer (6, 7).

Bacterial cells exist in a complex consortium of ecological interactions that may influence the functional profile of the microbial community, as well as the host’s health. The physiological gut microbiome is mainly symbiotic and modulate host metabolism, immune response, cell signaling, pathogen colonization resistance, and mucosal regeneration. Thus, microbes form intricate associations within communities, leading to both direct and indirect interactions among their members (8, 9). Although the mechanisms that drive dysbiosis remain unknown, it is suggested that bacterial interactions can potentially promote or hinder tumor development (10–12).

Virulent bacteria are a significant environmental factor associated with an increased risk of CRC, as shown by studies that have focused on the relationship between CRC and diet (13), inflammation (14), and immune cell infiltrates in the colonic mucosa (15).

In the current study, our hypothesis was that dysbiosis of the gut microbiota could potentially serve as a biomarker for estimating the environmental additive risk of CRC in individuals with LS. Thus, first, we performed whole shotgun metagenomic analysis on fresh stool samples obtained from 17 families with LS. This analysis included 19 LS-CRC patients and 15 asymptomatic healthy first-degree relatives. In our study, we characterized the genera and species present in the gut microbiota and identified a distinct set of bacterial signatures between LS-CRC patients and first-degree relatives who were asymptomatic, where diet did not show significant difference. Next, we constructed bacterial networks. By comparing, the network structures, we observed significant differences in terms of correlation (co-occurrence designed as positive, co-exclusion, designed as negative) between CRC patients and controls.

To validate our findings, we conducted a prospective study involving 150 asymptomatic individuals with LS who were enrolled in a French national biennial follow-up colonoscopy program. Through our investigation of their fecal microbiota, we identified similar differential networks between patients with precancerous lesions and controls with a normal colonoscopy, supporting the consistency of observations from the pilot study. Furthermore, in both the pilot and validation cohorts, networks constructed based on bacteria-bacteria correlations showed the ratio of virulent/symbiotic co-exclusion as the primary differential network marker between patients and controls.

In the final stage of our study, we quantified the abundance of *Escherichia* and *Bifidobacterium*, selected as bacterial candidates for virulent/symbiotic ratios, respectively, using quantitative PCR (qPCR). We observed significant differences between patients and controls in the validation study. We quantified these two bacterial candidates, *Escherichia coli* (*E. coli*) and *Bifidobacterium longum*, in the fecal samples of 100 asymptomatic individuals using quantitative PCR (qPCR). This finding further supports the differential bacterial interactions observed in the main network and suggests a potential role of virulent/symbiotic ratio enhancement in the development of colonic tumors in individuals with LS. Hence, we conclude that in individuals with LS, an imbalance between virulent and symbiotic bacteria can potentially identify those at a higher risk of developing CRC. This increased risk may be attributed to environmental factors such as dietary habits. As a connection to the environment, a limited number of virulent bacterial species, albeit of low prevalence, become the core of network constructions and qPCR analysis. Based on these findings, we propose the utilization of gut bacteria quantification as an additional tool for estimating the risk of CRC in asymptomatic individuals with LS. This approach has the potential to enhance the early detection of tumors.

## Patients, materials, and methods

### Recruitment of participants and collection of samples

Symptomatic patients referred to academic hospitals in the Paris area for colonoscopy were enrolled in several prospective cohorts. Effluents and tissues from enrolled participants were utilized for various types of studies, including experimental, proof-of-concept, discovery, and correlation analyses. Additionally, a nutritional questionnaire was given to the participants for further characterization and information gathering. For a detailed description of the questionnaire, please refer to **Supplementary Table S1** and (8). For a comprehensive analysis of bacteria species in the current pilot study, fecal DNA samples from LS individuals were subjected to whole metagenomic analyses. The individuals were members of 17 LS families who are currently being followed at academic hospitals. This pilot cohort study (Acronym: Malys), included 19 CRC patients and 15 healthy first-degree relatives. The participants in the study were selected from LS families, specifically from the first-degree relatives of CRC patients. To be eligible for inclusion in the study, individuals had to belong to an LS family with a positive genetic test for any of the MMR genes. Patients presenting with cancer were included in the study if they had at least one healthy relative who could be identified and included in the study as well. Among the cases, there were 19 with MSI-H, 13 with hMLH1 mutation, 2 with hMSH2 mutation, 2 with hMSH6 mutation, 1 with PMS2 mutation, and 1 case with an undetermined mutation. All individuals received detailed information about the study, including its objectives and the samples they would need to provide.

The study received financial support from Canceropole Ile de France through its Emergence grant program in 2016. The study was also promoted by the National Institute of Scientific Research in Medicine (INSERM), registered under 04–2004, and revised as CPP-IDF IX-11-019 by the consultative ethical committee in the Ile de France-Est medical district and Assistance Publique Hopitaux de Paris-APHP.

The validation cohort study comprised 150 asymptomatic individuals with LS who underwent biennial colonoscopy for screening purposes. These individuals were enrolled in a prospective prevention low-dose aspirin trial (ClinicalTrials.gov *Identifier: NCT04791644*) after colonoscopy at baseline. Exclusion criteria in the current analysis were a history of colorectal surgery, familial adenomatous polyposis, gastroenteritis and/or inflammatory bowel disease. All participants received information in writing, and formal written consent was obtained in triplicate copies: the patient retained one copy, one copy was kept in the clinical research centre or departments (CIC or URC) of the different centres, and the third copy was retained by the overseeing agency (Assistance Publique Hopitaux de Paris). Biological samples were obtained before colonoscopy and/or surgery and/or chemotherapy with no exposure to antibiotics during and/or colonic washing procedure the 3 weeks preceding the fecal sampling. The colonoscopy and pathology files were registered. The samples were stored at -80°C until their use.

### Food habits and intake through an auto questionnaire

To relate the food habits to metagenomic data, we asked individuals to complete a questionnaire on nutrition and food intake before the colonoscopy. Using this questionnaire, we estimated the daily quantities of various components in food and drinks based on the methodology described by Touvier et al. (16) (**Supplementary Table S2**).

### Human fecal DNA extraction

Fecal samples were collected using two methods: either in a sterile flask at the hospital or with the OMNIGENE-GUT kit (Ref OM-200) distributed by DNA GENOTEK (Palladium Drive Ottawa, Ontario, Canada K2V 1C2, Subsidiary of OraSure Technologies, Inc.) for home collection. Once collected, the samples were frozen at -80°C for preservation.

All frozen fecal samples underwent total prokaryote DNA extraction. For metagenomic analysis, DNA was extracted for prokaryote DNA characterization by using to GNOME® DNA Isolation Kit (MP Biomedicals, Santa Ana, CA) as described elsewhere (9).

For 16s rRNA analysis, the QiAamp DNA stool Mini Kit (Qiagen, Courtaboeuf, France) was used, following the instructions provided by Qiagen. The DNA was quantified using the Qubit® instrument (ThermoFisher Scientific, Montigny-le-Bretonneux) and then stored at -20°c until further sequencing and/or qPCR measurements. The DNA Extracts underwent sequencing either entirely (deep whole metagenomic in the pilot study) or after V3-V4 region amplification (for 16s rRNA targeted region and qPCR). The sequencing was performed using the Illumina MiSeq platform (San Diego, California · USA), as previously described in references (17, 18).

### Real-time polymerase chain reaction

In the Lab the PCRs were conducted using an ABI 7000 Sequence Detection System device and a standard range, based on varying quantities of *E. coli* DNA, was performed after DNA extracts from pure bacterial cultures; Ct curve has been calibrated based on CFU. This was done to enable the absolute quantification of bacterial DNA referred to as “All bacteria” in samples, as well as two bacteria of interest identified through bioinformatics analysis of bacterial sequences.

The qPCR reactions were executed in a final volume of 25 μl, comprising 1 ng of fecal DNA and 200 nM of each forward and reverse primer (Applied Biosystems, Waltham, Massachusetts, USA). A literature review, along with our own and other research (17–20), enabled us to pre-select several pairs of bacterial primers. The chosen primer pairs (**Supplementary Table S7**) were procured from Applied Biosystems® (Waltham, Massachusetts, USA).

In this study, we quantified the abundance of all bacteria (All Bacteria) and two bacterial genera (*Bifidobacterium* and *Escherichia coli*). The levels of these genera were found to be correlated with colonoscopy data.

### Taxonomic profiling of fecal microbiota

Taxonomic profiling from Whole Metagenome Sequencing was performed as follows: sequencing was done using a 100-bp single-end sequencing protocol on the Illumina MiSeq platform. The raw FASTQ files obtained from sequencing were subjected to quality-filtered using Trimmomatic (sliding windows of 2 bases with a quality score of 28) and high-quality reads were mapped onto the non-redundant protein database using Diamond (v0.9.17) (21) and MEGAN6 (v6.12.0) (22).

Taxonomic profiling from 16s rRNA sequencing was performed as follows: after amplification by PCR of the V3-V4 region of the 16s rRNA gene, sequencing was done using a 250-bp paired-end sequencing protocol on the Illumina MiSeq platform. We used the DADA2 pipeline (with default parameters except for the truncation length set to 245 bp) with the SILVA database non-redundant SSU database (v138.1) (23).

We used the **S**Hiny **A**pplication for **M**etagenomic **AN**alysis (SHAMAN: https://shaman.pasteur.fr/) server (24) for genus summarization, taxonomical exploration, Principal Component Analysis and differential abundance analysis. SHAMAN is based on the R software and implement the generalized linear model (GLM) approach from the DESeq2 R package (25) to detect differences in the abundance of species between two sub-groups. In our analysis, we defined a GLM that included gender, age, BMI and group as main effects. Differences (between genus and species) associated with adjusted *p*-value for multiple testing less than 0.05 were considered significant.

### Enterotypes of samples

Enterotypes were constructed according to Arumugam et al. (26).The enterotypes were inferred from the genus abundance tables using the classifier developed by Costea et al. (27). The classification was performed using their website at https://enterotype.embl.de/ (access September 2019), and defined as: “Enterotype ‘ET_B’ has *Bacteroides* as its best indicator; Enterotype ‘ET_P’ is driven by *Prevotella* and Enterotype ‘ET_F’ is distinguished by an overrepresentation of *Firmicutes*, most prominently *Ruminococcus*.

### Construction of bacteria networks

To further advance in network construction, we extracted a sample-taxa matrix (designed at a genus level) for each subgroup, with comparisons as follow: CRC patients versus first-degree relatives; asymptomatic LS individuals with normal versus precancerous lesion at colonoscopy. Then, we used the FastSpar/C++ implementation of the SparCC algorithm to estimate the correlation between pairs of taxa (based on their abundances) for microbial network inference (28, 29). Briefly, the SparCC algorithm computes the Pearson correction between the centred log-ratio (CLR) of taxa abundances. Correlation between two associated bacteria throughout all bacteria were considered significant for a *p*-value < 0.05. Subsequently, the statistical significance of the inferred correlations was evaluated through a bootstrap procedure. In this study, networks were constructed based on correlation coefficients ranging from -1 to -0.3 (co-exclusion) and +0.3 to +1 (co-occurrence).

In the initial set of analyses networks were independently constructed for each cohort, regardless of the prevalence of the genus within the cohort.

We used the Cytoscape software (30) for visualizing the networks. The edges were color-coded as follows: red was used for positive correlations (indicating co-occurrence) and blue was used to represent negative correlations (indicating co-exclusion). The size of each node in the network visualization was set to be proportional to the average abundance of the corresponding bacterium in the studied population.

To mitigate the potential influence of false correlations caused by low-prevalence bacteria, we conducted a thorough examination of the prevalence of bacteria within the cohorts depending on the size of each cohort. In the pilot study, we specifically focused on correlations between bacteria that were prevalent in at least 30% of the patient population.

In the validation study utilizing 16s rRNA datasets, we focused on correlations between bacteria that were prevalent in at least 50% of the population.

This selection criterion aimed to highlight virulent bacteria, oral bacteria, and symbiotic bacteria that demonstrated higher prevalence and potentially greater significance in the context of the study (31).

We employed *in-house* Python programs for several tasks, including the computation of the prevalence of each genus or species, data management, as well as the number of nodes, co-occurrences and co-exclusions in the networks.

## Results

**Figure 1:**
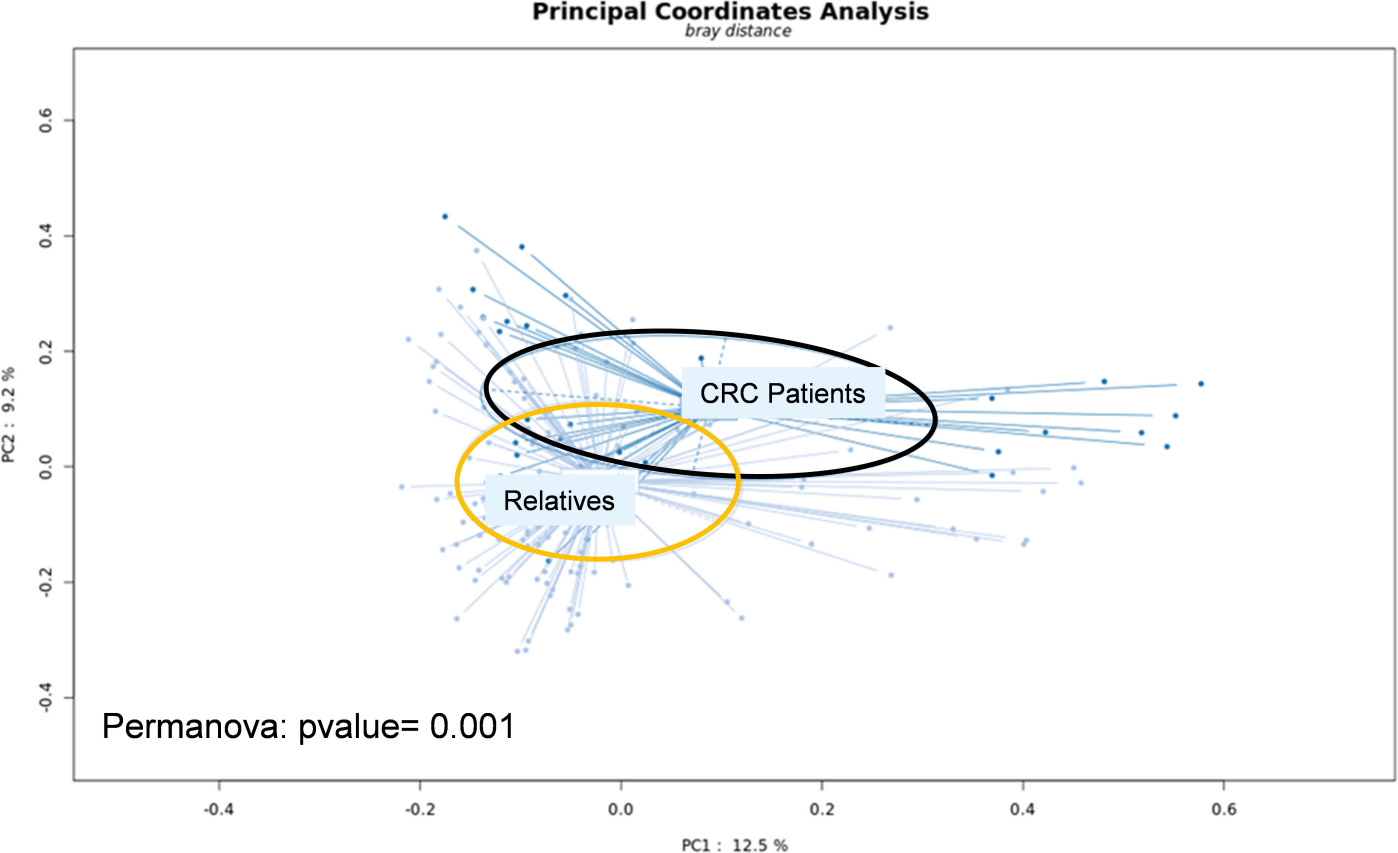
Fresh stool samples were collected prior to colonoscopy, surgery and/or chemotherapy. Bacterial DNA was extracted from the collected stools and submitted to whole-genome shotgun sequencing. After trimming the reads, they were aligned to the non-redundant protein database (nr) using the Diamond software. Taxonomical profiling was obtained using MEGAN6. Principal Coordinate Analysis (PCoA) was performed at the genus level using the SHAMAN website.

The main characteristics of CRC patients in the pilot study were comparable to their first-degree relatives except for age and presence of intestinal-associated disease (**Table 1**). At the genus level, the analysis of the whole metagenome demonstrated a significant separation between LS-CRC patients and healthy LS first-degree relatives, as assessed by PCoA analysis (**Figure 1**).

**Table 1:** Characteristics of the patients in the pilot study, Lynch syndrome CRC cases and first-degree relatives (n=34).

	Control subjects - First degree relatives	CRC Patients	*p-*value
n	15	19	
Age, years, mean ± SD	57.1 (16.48)	58.2 (13)	0.05
M/F gender, n (%)	9/6	10/9	0.2
BMI, kg/m², mean ± SD	23.75 (4.27)	24.7 (1)	0.5
History of Polyps Colorectal cancer Other cancer or disease*	0 0 1	6/19 17/19 5/19	0.001 0.001 0.05
Familial history Polyps in First degree relatives CRC in First degree relatives	Yes Yes	Yes Yes	NA
Comorbidity Diabetes, n (%) Hypercholesterolemia, n (%)	2 (13) 1 (7)	3 (16) 2 (10)	0.19 0.20
Any long-term treatment, n (%) **┴**	3 (20)	5 (26)	0.75
Nutrition Diabetes or other comorbidity, n (%)	2 (13)	3 (16)	0.19
Tumor location, staging Rectum Left colon Right Colon Stage I-II Stage III-IV	NA NA NA NA NA	1 4 14 2-5 11-1	NA

From September 2004 up to December 2016, 19 LS-CRC cases, 4 of them presenting with additional colonic adenomatous and 15 LS healthy individual without cancer and/or colonic polyps were selected from 17 LS families. To be included, they had to belong to a LS family with a positive gene mutation test in any of the MMR genes. Patients presenting with cancer were included if they had at least one healthy first-degree relative who could be identified and included.

**┴** Antibiotic therapy was excluded. Stool samples were collected prior to the colonoscopy; fecal DNA was extracted and subjected to a sequencing procedure. IBD refer to Inflammatory Bowel Disease.

*An IBD and five cases of cancers other than colonic cancer (including melanoma, gastric, cervical and bladder locations) were observed. NA, not applicable.

The statistical analysis (including age, BMI, and gender as covariables) revealed significant differences in genus-level bacteria abundances. Five genera were found to be significantly different. Specifically, virulent genera such as *Escherichia*, *Enterobacter*, *Thioalkalimicrobium* and *Klebsiella* were found to be more abundant in the stool of CRC patients compared to their healthy relatives; conversely, the *Butyrivibrio* genus showed a lower abundance in CRC patient (**Table 2**). Furthermore, several species within these genera were identified as significantly different (See **Supplementary Table S3** for details).

**Table 2:** Genera as identified between CRC patients and first-degree relatives in LS families.

Id	Base Mean	log2 Fold Change	*p-*value adjusted
*Escherichia*	932.26	-8.068	0.0005
*Enterobacter*	97.93	-6.779	0.0152
*Thioalkalimicrobium*	33.89	-6.869	0.0152
*Butyrivibrio*	2055.15	5.167	0.0243
*Klebsiella*	208.73	-6.061	0.0454

In addition to the differentially abundant genera mentioned earlier, there were trend towards significance in differences between CRC patients and relatives for 20 additional genera. These included *Desulfurivibrio, Shigella, Solobacterium, Peptostreptococcus, Prevotella, Paraprevotella, Bacteroides, Akkermansia, Streptococcus, Bifidobacterium, Alistipes, Roseburia, Blautia Eubacterium, Faecalibacterium, Gemella, Roseburia, Thermodesulfovibrio, Thioalkalimicrobium, Ruminococcus (* **Supplementary Figure S2**).

## Association between food habits and fecal symbiosis

Members from 4 out of 17 LS families reported having dietary habits that could be characterized as unique or specialized cuisine choices. These LS individuals belonged to families C1 (of Chinese origin; two members), C2 (of Lebanese origin, 3 members), C4 (of Portuguese origin, 3 members), and C5 (of Moroccan origin, two members) each of which had at least one CRC case. The remaining cases, consisting of 24 individuals (including 15 CRC patients), were included in the analysis for family C3 (three members, 2 CRC patients) as no specific dietary habits could be estimated for them. It has been suggested to group human gut microbiota compositions into three main compositional categories denoted enterotypes (26) based on a relatively high abundance of *Bacteroides* spp. (Enterotype 1 or ET-B), *Prevotella* spp. (Enterotype 2 or ET-P), or *Ruminococcus* (Enterotype 3 or ET-F). As one-third of CRC cases in LS families were found to show Enterotype 3 based on metagenomic analysis, we decided to analyze their dietary habits more deeply.

Overall, despite observing a tendency for differences in dietary habits between related individuals with and without CRC, we did not find statistically significant differences, possibly due to the small sample size of the present series and large number of food items. Nevertheless, the analysis of enterotypes showed *Ruminococcus* to be less common in LS-CRC as compared to healthy first-degree relatives, along with trends toward higher protein and salt intake, as assessed by the nutritional questionnaire. These observations were consistent with CRC patient microbiota enrichment in virulent bacteria such as *Enterobacter* and *Escherichia.* These genera likely benefit from salted and sulfured nutrients. Moreover, symbiotic butyrate-producing bacteria such a*s Faecalibacterium, Dorea*, and *Roseburia* were co-linked and diminished in LS-CRC patients compared to their healthy first-degree relatives (*p*-value <0.01, **Supplementary Figure S4**), although there was overlapping of the abundant bacteria in various families (**Supplementary Table S5**).

Significant differentially abundant genera or those with a trend toward significance were detected across all CRC enterotypes as compared to healthy relatives, with *Escherichia* showing greater enrichment in CRC exhibiting enterotypes 2 and 3 (ET_P and ET_F) (**Supplementary Figure S2**). Overall, CRC-linked clusters that included *Eubacterium, Faecalibacterium*, or *Ruminococcus* had significant decreases associated with at least one virulent bacterium.

The principal coordinate analysis (PCoA) of all samples also demonstrated strong clustering into three enterotypes. Even the Enterotype 1 (ET_B) contained a high proportion of *Ruminococcus* and *Faecalibacterium* (≥40% of all genera, with more *Ruminococcus*), (**Supplementary Figure S1**; **Supplementary Table S4**), making the distribution of gut microbiota into known enterotypes (1 to 3) less informative regarding the food habits of individuals.

**Figure 2:**
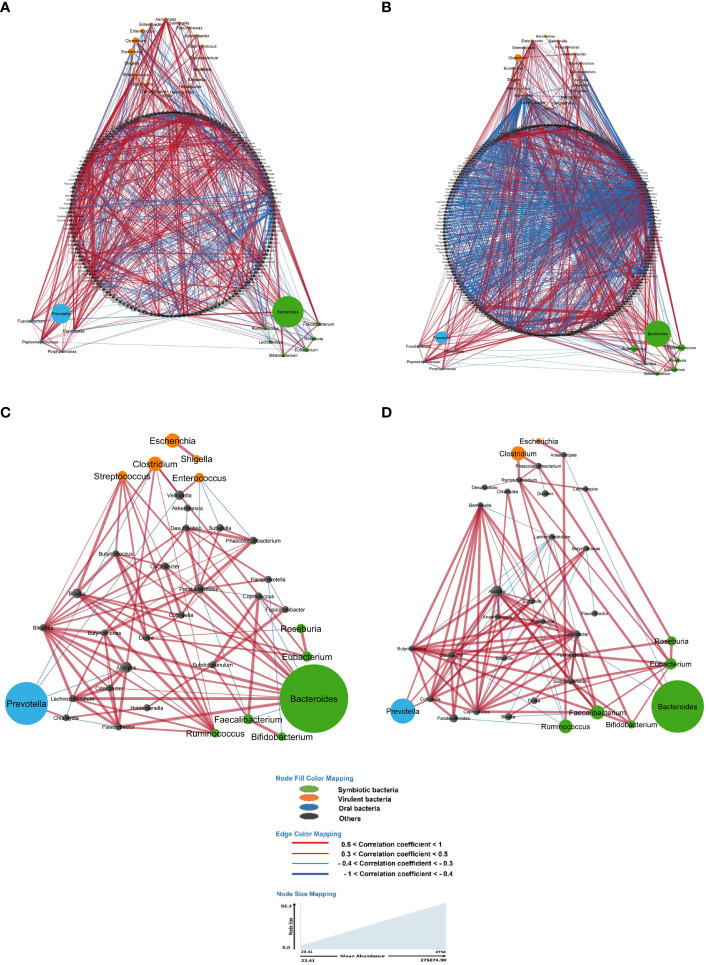
In the gut microbiota analysis of LS-CRC patients **(A, C)** and their healthy first-degree relatives **(B, D)**, networks were constructed based on significant bacterial correlations. In Figures **(A, B)**, all bacteria, regardless of their prevalence, were included in the network construction. However, in Figures **(C, D)**, only bacteria with a prevalence of 30% or more were included. In these network visualizations, each node represents a bacterium, and the size of the node is proportional to its average abundance in the studied population. The edges between nodes represent correlations with other bacteria, where colors design the direction of the correlation; red indicates positive correlations (co-occurrence) and blue indicates negative correlations (co-exclusion). The width of the edges corresponds to the value of the correlation coefficient, reflecting the strength of the correlation between bacteria.

Particularly, the main genera involving these associations included *Bacteroides, Clostridium, Coprococcus, Dorea, Eubacteria, Faecalibacteria, Prevotella, Ruminococcus and Roseburia* with strong co-variation in LS-CRC individuals (**Figure 2** *;* **Supplementary Figure S3**). Although four genera (*Ruminococcus, Faecalibacteria, Eubacteria* and *Clostridium)* remained abundant in both CRC patients and healthy first-degree relatives, a significant imbalance is observed in CRC patients as compared to first-degree relatives (**Supplementary Figure S3**).

Taking all individuals together, the importance of food habits in the bacteria networks was noticed, as more than 1/3 of CRCs in LS families were found in the group 3 Enterotype (ET-F, G3). Even in this Enterotype subgroup, *Faecalibacterium* and *Ruminococcus* were lower as compared to healthy LS relatives. Accordingly, correlations (co-occurrence and co-exclusions) in LS-CRC patients with these genera were characterized, and the results are given in **Supplementary Data** (**Supplementary Figure S4**).

## Bacteria network

When considering all bacteria, the comparison of bacterial networks between LS-CRC patients and their healthy LS first-degree relatives (controls) showed a significantly lower number of correlations, including both co-occurrences and co-exclusions, in CRC patients (**Figure 2**; **Table 3**). Differences in the number of correlations between LS-CRC patients and healthy relatives were found to be statistically significant (*p*-value < 2.2 * 10^-22^). Additionally, the ratio of co-occurrences to co-exclusions was found to be two times higher in LS-CRC patients compared to healthy relatives. When the dataset was filtered according to a prevalence of less than 30% in the cohort for each genus, this difference was no longer significant (**Table 3**, *p*-value = 0.6625). This suggests the importance of low prevalent genera and/or bacteria functions in the network construction.

**Table 3:** Correlations through network of bacteria in LS-CRC patients and their 1st-degree relatives.

	Number of samples	Number of nodes	Number of correlations	Co-occurrences	Co-exclusions	Co-occurrences/ Co-exclusions	Co-occurrences/Total	Co-exclusions/ Total
**LS-CRC, all bacteria (** **Figure 2A**)	19	216	1052	666	386	1.72	0.63	0.37
**F1st-degree relatives, all bacteria (** **Figure 2B**)	15	275	1848	814	1034	0.79	0.44	0.56
**LS-RC, prevalence 30% or more (** **Figure 2C**)	19	35	99	85	14	6.07	0.86	0.14
**1st-degree relatives, prevalence 30% or more (** **Figure 2D**)	15	35	104	87	17	5.18	0.84	0.16

## Bacterial networks and imbalance “pro inflammatory/symbiotic bacteria” in the validation cohort

To validate bacteria networks and related imbalance between pro-inflammatory and symbiotic bacteria, we conducted a second study involving 150 asymptomatic LS individuals. Colonoscopy and histopathology assessments of biopsies were performed, resulting in the identification of 79 patients with precancerous lesions (such as flat and/or polyp adenomatous) and 71 patients with various types of lesions. The characteristics of these patients are summarized in **Table 4**.

**Table 4:** A. Characteristics of the asymptomatic individuals submitted to fecal 16S rRNA sequencing, N = 150.

	With event (Yes)	Without event (No)	*p*-value
**Colonoscopy**	N= 79	N=71	–
**Age (mean ± SD)**	48.99± 9.60	47.55 ± 12.68	0.44
**Gender, M: n (%)**	39	34	0.99
**Gender, F: n (%)**	40	37	0.99
**BMI: (mean ± SD)**	25.22 ± 3.99	25.16 ± 4.13	0.97
**Height: (mean)**	171.41 ± 9.06	170.09 ± 9.83	0.35
**Weight (mean ± SD)**	73.40 ± 14.37	73.20 ± 14.54	0.68

SD, standard deviation; BMI, body mass index; M, Male; F, Female.

**Table:** B. Characteristics of the asymptomatic individuals submitted to qPCR analysis, N = 100.

	With event (Yes)	Without event (No)	*p*-value
**Colonoscopy**	N= 51	N=49	–
**Age (mean ± SD)**	47.98 ± 9.93	45.77 ± 12.82	0.34
**Gender, M: n (%)**	19	26	0.99
**Gender, F: n (%)**	32	23	0.99
**BMI: (mean ± SD)**	24.99 ± 3.98	25.23 ± 4.03	0.76
**Height: (mean)**	170.85 ± 8.98	171.25 ± 9.82	0.83
**Weight (mean ± SD)**	73.05 ± 13.31	74.59 ± 12.01	0.62

SD, standard deviation; BMI, body mass index; M, Male; F, Female.

Overall, 150 individuals with a constitutional gene mutation conferring an increased risk for neoplastic lesion occurrence (such as flat and/or polyp adenomatous) in the colon were including in the analysis (A); quantification of selected bacteria was performed on a subset of the cohort (B). They were divided into two groups based on the presence or absence of precancerous lesions observe during colonoscopy (Yes, No). There were no significant difference between these two groups in terms of age, gender, or BMI.

In the comparison of bacterial populations based on 16s rRNA sequences, two subgroups were formed according to colonoscopy and pathology results: those without lesion at colonoscopy (No) and those with precancerous lesions (Yes) as indicated in **Table 4A**. Analysis of fecal prokaryote DNA reads revealed the most abundant bacteria phyla in the whole gut bacterial populations of patients with colonic lesions, included *Actinobacteria, Firmicutes, Verrucomicrobia, Tenericutes, Fusobacteruim, Proteobacteria, Bacteroidetes, Spirochaetes, Elusimicrobia, Lentisphaerea* and *Synergistetes* (**Supplementary Figure S7A**). There was a trend towards higher abundances of *Firmicutes* and *Actinobacter* in individuals without colonic lesion (No). Several genera including *Lactobacillus, Turicibacter, Enterococcus, Clostridium, Haemophilus, Clostridium innocuum group, Veillonella, Lachnoclostridium, Paenibacillus, Ruminococcus gnavus* and *Bifidobacterium* were identified as differential (**Supplementary Figure S7B**). Of note, *Bifidobacterium* was found to be diminished in patients with a precancerous event at colonoscopy.

Indeed, a lower number of nodes and co-occurrences were observed in asymptomatic individuals with precancerous lesions at colonoscopy compared to those with a normal colonoscopy (**Table 5**, *p*-value = 0.01687). However, there were no significant differences regarding co-exclusions between the two groups. These differences remained significant even when the analysis was limited to bacterial populations with a prevalence of 50% or more (**Table 5**, *p*-value = 0.00726).

**Table 5:** Descriptive statistics of correlation network of fecal bacteria in LS asymptomatic individuals according to precancerous lesion (No vs Yes) at colonoscopy.

	Number of samples	Number of nodes	Number of correlations	Co-occurrences	Co-exclusions	Co-occurrences/ Co-exclusions	Co-occurrences/Total	Co-exclusions/ Total
**LS YES (** **Figure 3A** **), all bacteria**	79	140	760	539	221	2.44	0.71	0.29
**LS NO (** **Figure 3B** **), al bacteria**	71	146	824	628	196	3.20	0.76	0.24
**LS YES (** **Figure 3C** **), prevalence 50% or more**	79	58	98	70	28	2.5	0.71	0.29
**LS NO (** **Figure 3D** **), prevalence 50% or more**	71	64	128	110	18	6.11	0.86	0.14

**Figure 3:**
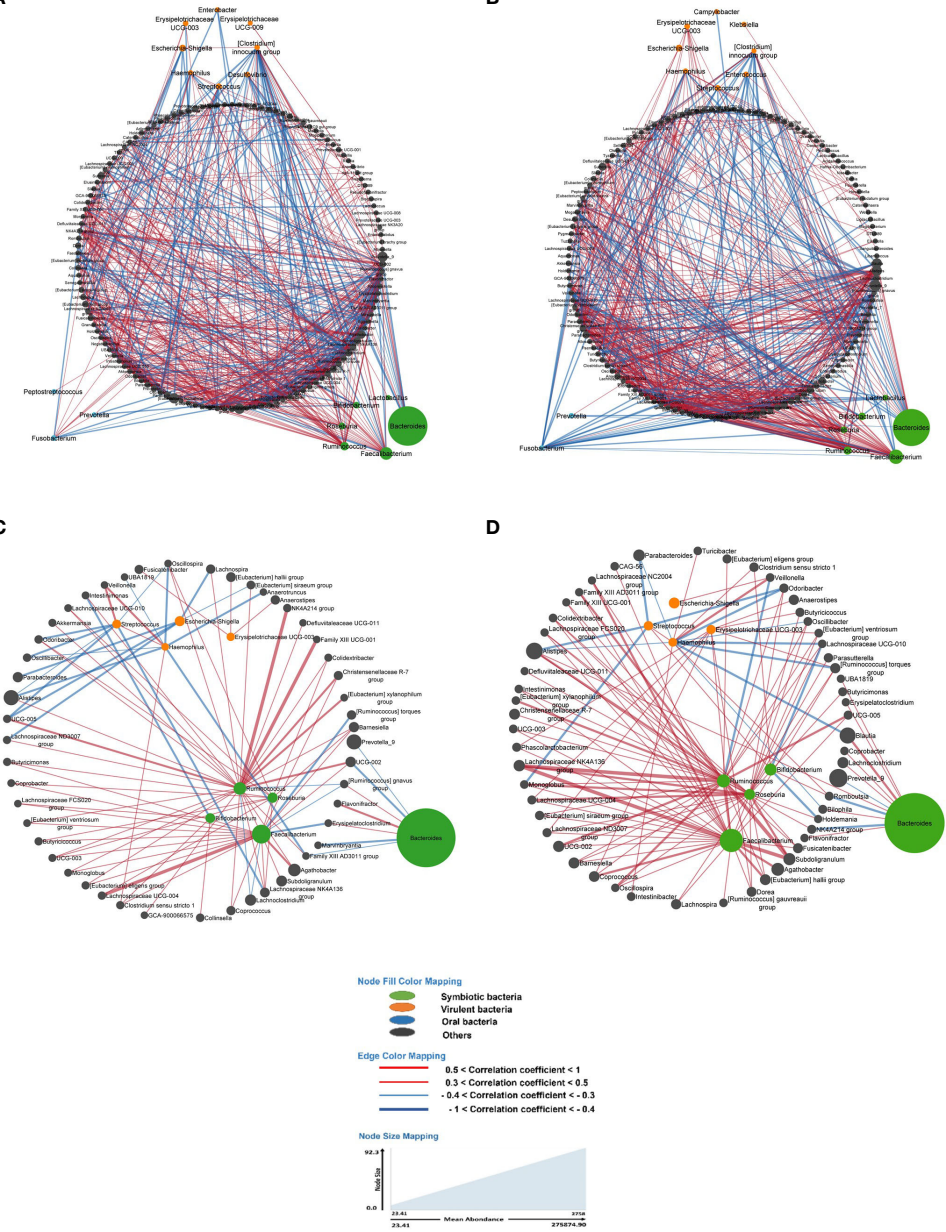
In all figures, each node represents a bacterium, and its size is proportional to its average abundance in the studied population. The edges between the nodes represent correlation of the relative abundance of the bacteria. Positive correlations (co-occurrence) are shown in red, while negative correlations (co-exclusion) are shown in blue. The width of the edges indicates the value of the correlation coefficient, providing an indication of the strength of the correlation. **(A, B)** depict the bacterial networks of fecal bacteria in LS individuals presenting with and without precancerous lesions during colonoscopy, respectively. In both figures, all bacteria were included in the analysis. **(C, D)** represent the bacterial networks in LS individuals with and without precancerous lesions, respectively. These figures include only those bacteria with a prevalence of 50% or more.

The analysis of the main core of the bacteria network revealed *Escherichia* as a pro-inflammatory bacterial genus clustered with several other bacteria, including *Lachnospira*, *Lachnospiraceae ND3007 group*, *[Eubacterium] siraeum group*, *[Clostridium] innocuum group*, *UCG-005* and *Aquamonas* (**Figures 3A, C**) in asymptomatic LS individuals with precancerous lesion at colonoscopy compared to individuals with a normal colonoscopy. Furthermore, in individuals with a normal colonoscopy, the symbiotic bacterium *Bifidobacterium* was found to be higher in abundance and clustered together with *Romboutsia*, *Streptococcus*, *Clostridium sensu stricto 1, Lachnospiraceae UCG-010* and *Holdemania* (**Figures 3B, D**). These genera were significantly associated with various correlation coefficient (CC) with *Escherichia.*

Briefly, the bacterial correlation network in asymptomatic individuals’ gut microbiota was clearly different in those with precancerous lesions as identified during colonoscopy (Yes) compared to those with a normal colonoscopy (No) in terms of the number of nodes as well as the pattern (**Figure 3**).

As main examples, in LS asymptomatic individuals with precancerous lesions at colonoscopy, the virulent bacterium *Escherichia* displayed negative correlations (co-exclusions) with bacteria such as [*Eubacterium*] *siraeum group* (CC= -0.4), *Lachnospira* (CC= -0.31), *Parabacteroides* (CC= -0.32), *Lachnospiraceae* ND3007 group (CC= -0.34), and UCG-005 (CC= -0.37). *Additionally*, *Escherichia* showed a positive correlation (co-occurrence) with [*Clostridium*] *innocuum group* (CC= +0.39) and *Aquamonas* (CC= +0.41) in this group (**Figure 3A**).

In individuals with a normal colonoscopy, *Escherichia* exhibited a positive correlation (co-occurrence) with *Veillonella* (CC= +0.35) and *Aquamonas* (CC= +0.36) (**Figure 3B**).

## Determination of virulent/symbiotic bacteria ratio using two bacteria candidates

**Figure 4:**
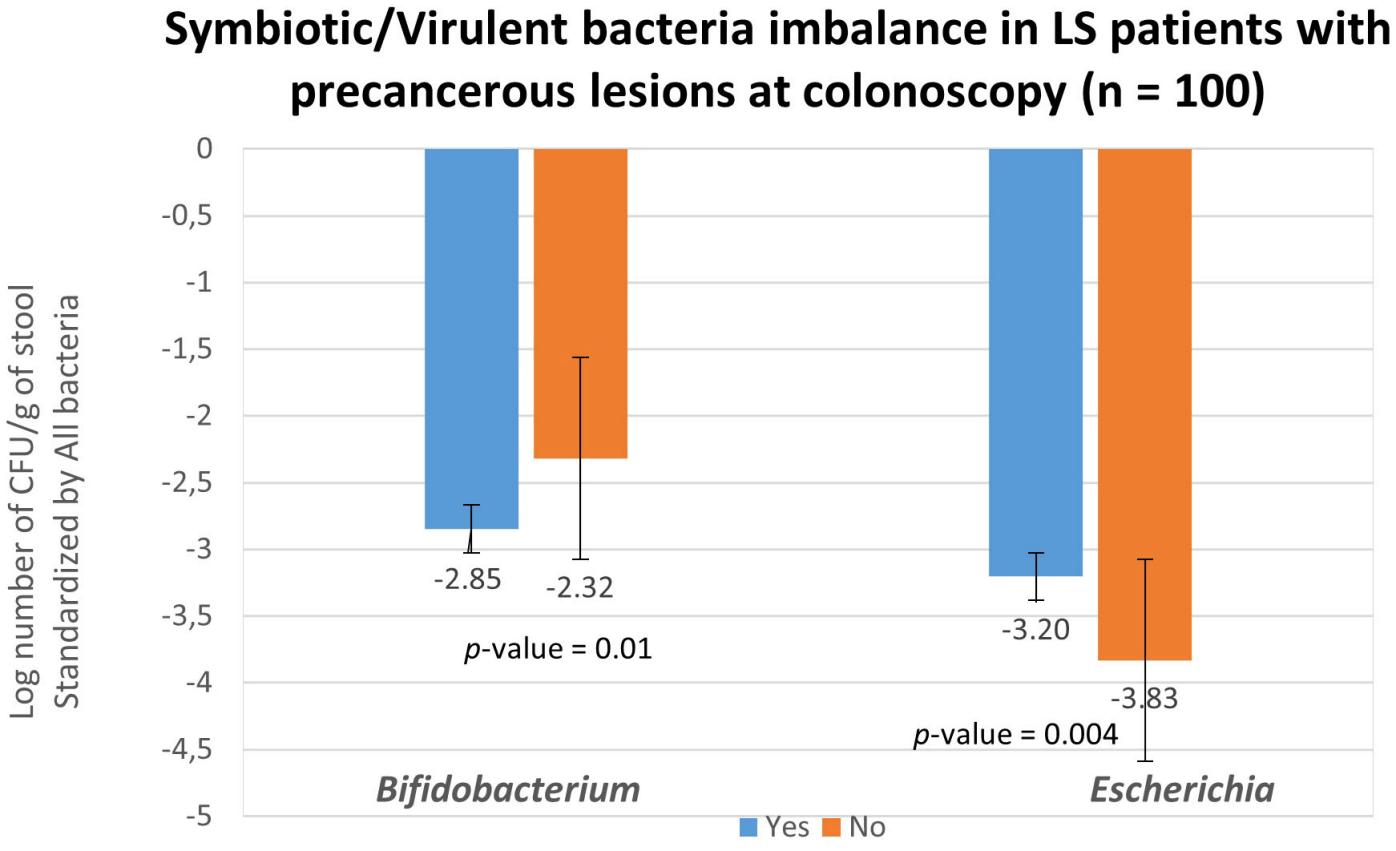
Fecal bacteria quantification in asymptomatic LS individuals according to colonoscopy and pathology findings. Abundances of all bacteria (represented by consensus sequences) as well as *Bifidobacterium* and *Escherichia* coli were determined using real-time qPCR with targeted primers in 51 individuals with precancerous lesion (blue; designed as Yes) and in 49 without precancerous lesion (orange; designed as No) observed during colonoscopy. The quantification of *Bifidobacterium* and *Escherichia* coli levels was normalized and reported as log (*Bifidobacterium* -all bacteria and *Escherichia* -all bacteria) per gram of stool. These values were normalized with respect to the quantity of all bacterial DNA present in the samples.

To validate the role of bacterial candidates as designed as virulent versus symbiotic, quantitative polymerase chain reaction (qPCR) was used in a subset of 100 patients (49 with no precancerous lesions: No vs 51 with precancerous lesions: Yes) for quantification, as outlined in **Table 4B**. Based on the qPCR quantification results, *Bifidobacterium* levels were significantly lower, while *Escherichia* levels were significantly higher in individuals with precancerous lesion in the colon compared to individuals with a normal colonoscopy (**Figure 4**) confirming the findings. The estimated abundance of *Escherichia* and *Bifidobacterium* in the analysis individuals’ feces as assessed by 16s rRNA sequence analysis was validated using qPCR.

## Discussion

The analysis of gut microbiota DNA revealed significant dysbiosis in CRC patients compared to their first-degree relatives with LS. Additionally, we demonstrated alterations in the organization of bacterial networks in CRC patients. In a validation cohort, we found that the bacterial network in asymptomatic LS individuals with precancerous lesions (polyps and/or flat adenomatous lesions) during colonoscopy was significantly different from that of LS asymptomatic individuals with a normal colonoscopy. In both cohorts, virulent bacteria at the core of the main network were negatively correlated with symbiotic bacteria. For instance, a higher abundance of *Escherichia* and a lower abundance of *Bifidobacterium* characterized the main network in LS asymptomatic individuals with precancerous lesions during colonoscopy. These results suggest that during the early stages of colon carcinogenesis, there is a depletion of anti-inflammatory symbionts, such as *Bifidobacterium*, in the stool, while there is an enrichment of virulent genera such as *Escherichia*, which is associated with pro-inflammatory properties. This dysbiosis, characterized by an imbalance between virulent pro-inflammatory harmful bacteria and symbiotic anti-inflammatory bacteria, enhances the risk of cancer development in individuals with LS.

A similar dysbiosis within the colonic microbiota has been previously reported in familial adenomatous polyposis (FAP), a condition that predisposes individuals to CRC. In FAP, colibactin-producing *Escherichia coli* and enterotoxigenic *Bacteroides fragilis*, both virulent species, were found to be overdeveloped in stool samples (32). The current alterations in bacterial networks within the gut microbiota as an early event in colon carcinogenesis are consistent with previous reports on the role of dysbiosis in the development and progression of colon cancer. This further suggests functional microbial interactions and associations rather than a direct impact of individual microbes. Notably, based on deeper sequencing of bacteria, virulent genera and species, including but not limited to *Escherichia coli* and *Bacteroides fragilis*, were found to be more abundant in the feces of CRC patients compared to their relatives and healthy first-degree relatives. Our current findings align with the results we previously reported in homozygous twin sporadic CRC patients (33) and with studies conducted by others in the context of familial adenomatous polyposis (FAP) (32–34). Particularly, the enrichment of virulent species in conditions augmenting the risk of CRC, such as IBD (35, 36) and FAP (17). Various mechanisms, including DNA breaks and/or epigenetic DNA changes caused by various virulent bacteria, including *Escherichia coli* (11, 37), have been suggested.

Furthermore, imbalances in the gut microbiota have been observed in individuals shortly after transitioning from African diets, which are typically rich in vegetables and low in fat and animal proteins, to American diets, which often have higher ratios of sugar, salt, fat, and animal proteins (38). These findings suggest that food habits may play a significant role in influencing gut dysbiosis. Since virulent bacteria, as the main core of the bacterial network, interact with symbiotics whose abundance decreases in CRC patients and individuals with precancerous lesions in the colon, one should identify factors that imprint such a chronic effect on bacteria clustering.

The first attempt consisted in characterizing gut enterotypes through bacteria clustering with diet (26). Enterotypes are distinct microbial community structures that have been identified based on the abundance of certain genera in the gut and the pattern they exhibit with co-associated bacteria through networks. *Prevotella* and *Bacteroides* are two predominant genera that characterize different enterotypes. The *Prevotella*-dominated Enterotype is often associated with high-fiber diets, while the *Bacteroides*-dominated Enterotype is more commonly found in individuals with diets higher in animal proteins and fats. In our study, we explored the enterotypes and identified a *Prevotella*-dominated Enterotype (ET_P or G2) that was associated with LS-CRCs. Interestingly, we observed that 68% of healthy first-degree relatives had a different enterotype, namely the *Bacteroides*-dominated Enterotype (ET_B or G1) although one-third of CRC cases in LS families showed enterotype 3 gut microbiota that contains mostly *Ruminococcus*, a predominantly butyrate-producing genus. Additionally, LS-CRC patients were more likely to have the *Prevotella*-dominated Enterotype compared to their healthy first-degree relatives, suggesting a potential association between Enterotype composition and CRC development.

Although based on a validated French questionnaire regarding consumed diet (16, 39–41), we only observed trends in association with diet, e.g. fewer vegetables and higher meat intakes with dysbiosis. As expected, *Prevotella*-dominated enterotypes in LS-CRC patients were characterized by *Escherichia*-*Shigella* enrichment compared to their first-degree relatives, with significant decreases in symbiotic such as *Ruminococcucus, Faecalibacterium, Coprococcus* and *Enterococcus.* Interestingly, all these symbiotics have been found to be co-associated (**Supplementary Figure S4**). Various virulent genera, such as *Bacteroides, Fusobacterium, Salmonella, Escherichia*, and *Campylobacter* have been shown to be statistically higher in CRC patients’ stool than in first-degree relatives’ stool (38, 42). We found similar patterns in 4 out of 17 LS families in the present study who reported having more traditional native diets. Comparisons between these families and those under French diets highlighted *Akkermansia* as differential genus in family C1 (Chinese diet) or C4 (Portuguese diet) (**Supplementary Table S5**).

Additionally, prokaryote sequences for species assignment in the pilot study revealed that *Thermodesulfovibrio yellostoni* was the only species over-represented in LS-CRC patients compared to healthy LS relatives (**Supplementary Tables S3**, **S6**). This species resembles functionally *Thioalkalivibrium cyclium*, a bacterium we found significantly over-represented in sporadic CRC patients as compared to healthy age- and gender-matched healthy individuals (data not shown). Of interest, several functions such as sulfur-oxidation and autotrophic heavy metal metabolism are shared among all species within the *Thermodesulfovibrio* genus. However, the present species is not known as a natural inhabitant of the human colonic niche, and the lack of culture of this bacterium in the laboratory may constitute a bias for its identification.

Although we could not rule out cross-homology of unidentified prevalent bacteria in LS-CRC patients resembling *Thermodesulfovibrio*, this taxon illustrates the higher abundance of a thermophilic, methanogenic and salt-dependent bacterium in CRC patients. These microbes can survive in anaerobic, hypersaline and sulfate- enriched environments (43, 44). Hence, global metabolites derived from bacteria networks should be considered. About 10% of all metabolites found in mammalian blood result from bacterial networks (45), including those produced by microbes from exogenously consumed compounds, produced by the host and biochemically modified by gut microbes, and synthesized *de novo* by gut microbes. As an example, salt-enriched food habits in the French cuisine (16, 39), could result in the presence of the *Thermodesulfovibrio* genus, which was found within the network that included virulent bacteria such as *Escherichia, Klebsiella, Haemophilus*, and *Shigella* (**Figure 2**). This functional can be influenced by dietary habits.

The overabundance of pro-inflammatory virulent bacteria is accompanied by reduced abundances of butyrate-producing species (46), higher levels of DNA damage (such as DNA mutations or deletions and/or hypermethylation in the intestinal epithelial cells) (47, 48), and stimulation of epithelial proliferation in the mucosa (49). Interestingly, we also demonstrated that butyrate-producing symbionts were decreased also in asymptomatic individuals who developed early precancerous lesions. This suggests that an enhancements of the virulent/symbiotic bacteria ratio may favor the growth of neoplasia in the colon, as previously reported in obese individuals (50).

*Bifidobacterium* is one of the most important symbionts exerting a potential role in protecting the colonic mucosa. Based on murine model studies, *Bifidobacterium* has been shown to have a probiotic effect against chemical-induced tumors (51, 52). For example, *Bifidobacterium animalis* limits the anti-mutagenic activity of the carcinogen 2-amino-3-methylimidazo [4, 5-f] quinolone in mice (53). Additionally, *Bifidobacterium longum* and a *B. breve*, two strains of the *Bifidobacterium* genus, can protect DNA from chemical carcinogen-induced damage in a rat model (54). It can also exert an inhibitory effect on the growth of potential competitors by enhancing the production of antimicrobial substances (55, 56), This genus is known to function in mutualism with *Lactobacillus* by producing folates, which are the main source of the epigenetic pathway of DNA changes and are reported as a flexible mechanism through interaction with the environment (57). Indeed, folate deficiency increases the risk of tumor development and the failure of CD8 infiltrates within tumor tissues due to the hypermethylation of TOX and HIF1 genes (57).

To the best of our knowledge, this is the first whole metagenomic analysis of enterotype-based fecal microbiota in CRCs with reference to LS individuals with at high risk of CRC, showing the potentially protective effect of *Bifidobacterium*. Furthermore, bacteria networks rather than individual microbes are currently being considered in relation to the potential metabolites they may produce or alter in the blood. This study requires longitudinal confirmation, which is ongoing with follow-up of patients through a prospective randomized anti-inflammatory chemotherapy (versus placebo) study in healthy LS individuals (ClinicalTrials.gov *Identifier: NCT04791644)* with the development of colon neoplasia as the primary endpoint. This study involves screening individuals for dietary factors, aiming to determine whether certain foods might interfere with the occurrence of neoplasia through changes in the microbiota. *Bifidobacterium* per se, and/or *Lactobacillus* or the metabolites they may produce, could be validated as protective markers before their validation in a high-risk CRC occurrence setting, including LS asymptomatic individuals and chronic IBD patients.

## Data availability statement

The data for this study have been deposited in the European Nucleotide Archive (ENA) at EMBL-EBI under accession number PRJEB70916 for Shotgun Metagenomic data (https://www.ebi.ac.uk/ena/browser/view/PRJEB70916) and PRJEB70919 for 16S rRNA data (https://www.ebi.ac.uk/ena/browser/view/PRJEB70919).

## Ethics statement

The studies involving humans were approved by Ethical committee in the Ile de France-Est medical district and Assistance Publique Hopitaux de Paris-APHP- under the number 09-2016. The studies were conducted in accordance with the local legislation and institutional requirements. The participants provided their written informed consent to participate in this study. Written informed consent was obtained from the individual(s) for the publication of any potentially identifiable images or data included in this article.

## Author contributions

MS: Writing – original draft, Writing – review & editing, Data curation, Formal analysis, Methodology, Investigation, Software, Validation, Visualization. DM: Data curation, Methodology, Writing – review & editing, Formal analysis, Investigation, Software, Supervision, Validation, Visualization. EC: Writing – review & editing, Formal analysis, Investigation, Methodology. RB: Writing – review & editing, Data curation, Formal analysis, Funding acquisition, Investigation. KK: Writing – review & editing, Conceptualization, Funding acquisition, Validation. IS: Conceptualization, Formal analysis, Investigation, Methodology, Software, Supervision, Validation, Writing – original draft, Writing – review & editing, Resources, Visualization, Funding acquisition, Project administration.
