# Supplementary material for: Loss of symbiotic and increase of virulent bacteria through microbial networks in Lynch syndrome colon carcinogenesis
Source: Front Oncol. 2024 Feb 5;13:1313735. doi: 10.3389/fonc.2023.1313735 (PMC10876293; doi:10.3389/fonc.2023.1313735)
Supplement: Supplementary file 1 [file DataSheet_1.pdf]

Supplementary Files

Table S1. Estimated (gr) Self-administered 24 h dietary record in LS families CRC patients (n=19) and first-degree relatives (n=15)

Daily food intake	LS CRC Patients; Mean (SD)	1st-degree Relatives; Mean (SD)
Fruits	309 (210)	337 (210)
Vegetables	370 (211)	325 (185)
Meat, poultry, offal	139 (67)	108 (72)
Processed meat	59 (40)	60 (59)
Fish, seafood	140 (98)	105 (59)
Eggs	92 (71)	70 (51)
Bread, toasts	152 (61)	101 (59)
Potatoes	161 (70)	155 (60)
Pulses	80 (100)	95 (91)
Pasta, rice, semolina, and other starchy foods	131 (91)	112 (59)
Milk, dairy products* and dairy desserts	260 (163)	295 (139)
Fats and sauces	60 (41)	39 (27)
Pizzas, snacks/fast food	52 (29)	38 (21)
Soft drinks (without fruit/vegetable juice)	1230 (656)	1350 (530)
Alcoholic drinks	310 (211)	59 (31)
Breakfast cereals	56 (29)	170 (159)
Cakes, biscuits, pastries	110 (74)	95 (73)
Sugar and confectionery	51 (46)	39 (31)

Table S2. Estimated (gr) Daily nutrient intake by self-administered 24 h dietary record

	LS CRC Pts Mean (SD)	1st-degree Relatives Mean (SD)
Energy (kcal)	2152 (549)	2019 (601)
Protein (g)*	94 (31)	82 (31)
Carbohydrate (g)	229 (68)	227 (81)
Fat (g)	85 (28)	80 (32)
Saturated fat (g)	35 (15)	31 (17)
Monounsaturated fat (g)	28 (10)	26 (12·4)
Polyunsaturated fat (g)	14 (7·5)	12·7 (8·5)
Cholesterol (mg)	354 (202)	343 (186)
Dietary fibre (g)	26 (14)	39 (7)
Vitamin C (mg)	130 (83)	133 (88)
Thiamin (mg)	1·7 (1·6)	1.6 (0·80)
Riboflavin (mg)	2·1 (0·8)	2.2 (0·86)
Niacin (mg)	31 (13)	38 (7.7)
Pantothenic acid (mg)	6 (2·2)	7.8 (1.2)
Vitamin B6 (mg)	2·1 (0·8)	2·1 (0·9)
Folate (mg)	385 (167)	173·3 (84)
Vitamin B12 (mg)	9 (12)	6·7 (9)
Total vitamin A (mg RE)	1522 (2243)	2200 (1001)
Retinol (mg)	707 (212)	824 (709)
b-Carotene (mg)	4912 (4001)	4825 (4312)
Vitamin E (mg a-TE)	12 (6)	13 (9)
Vitamin D (mg)	3·1 (4·2)	3 (4)
Ca (mg)	1005 (389)	1400 (931)
Fe (mg)	16·2 (8·8)	13 (10)
Mg (mg)	539 (197)	277 (801)
P (mg)	1498 (489)	1547 (560)
Zn (mg)	12 (3·9)	11·9 (3·7)
K (mg)	4930 (1073)	3633 (713)
Na (mg)*	3416 (219)	3100 (910)

* Significant difference ($p<0.05$)

Figure S1. Enterotypes and alpha diversity analysis

a)

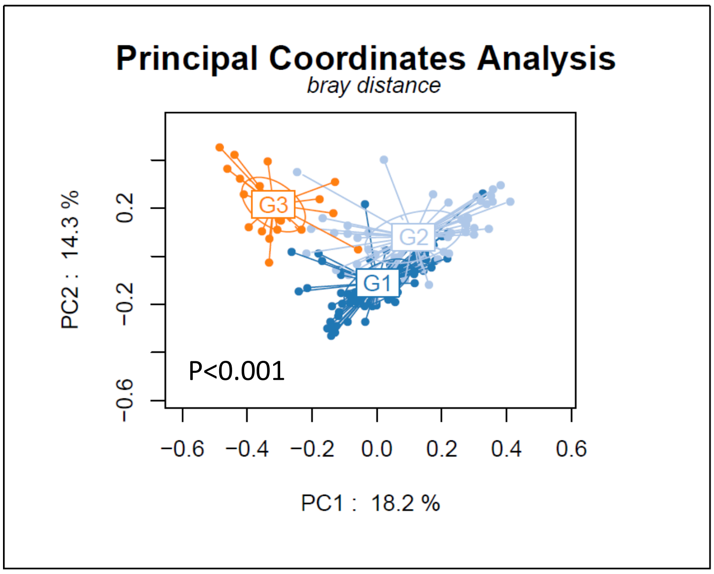

b)

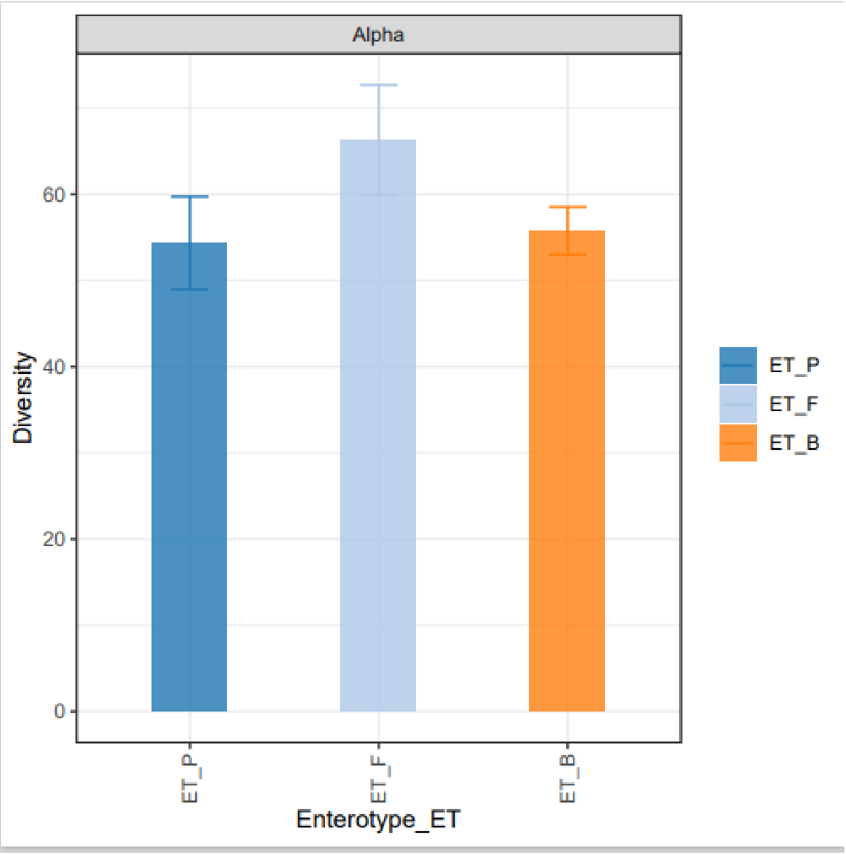

c)

Diversity	Var	value	ci.down	ci.up
Alpha	ET_P	54.34	48.95	59.73
Alpha	ET_F	66.31	59.96	72.67
Alpha	ET_B	55.76	53.02	58.51

a. *The PCoA plot represents the microbiota of all fecal samples, which were significantly different and clearly separated into the 3 Enterotypes: ET_B or G1; ET_P or G2); ET_F or G3.*
b. *Alpha diversity of Enterotypes in stool samples. Error bars indicate 95% confidence intervals.*
c. *Mean values of Alpha diversity regarding the Enterotypes in stool samples*

Figure S2

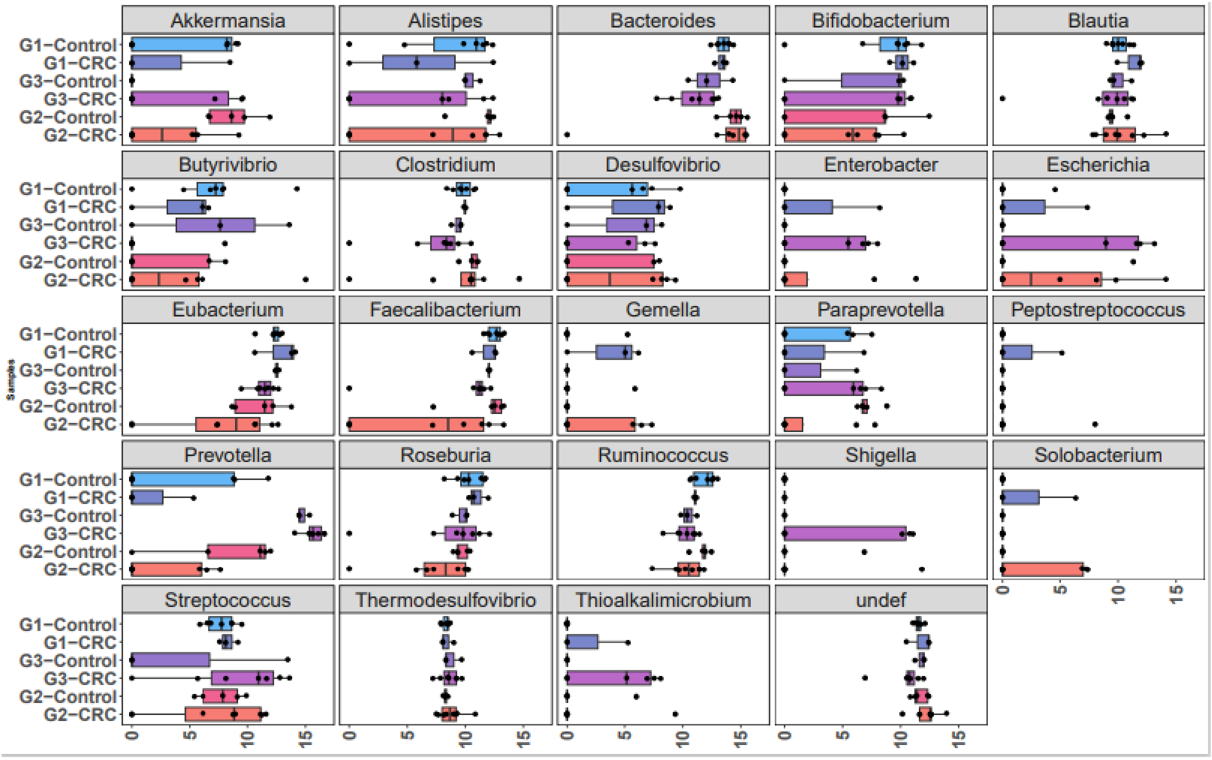

Differential abundant bacteria genera in three Enterotypes are indicated according to the individual values of bacteria in stool. CRC designs the patients with a colorectal cancer and Control designs first degree relatives.

Figure S3. Correlation clustering in LS CRC patients' feces as compared to 1st-degree relatives.

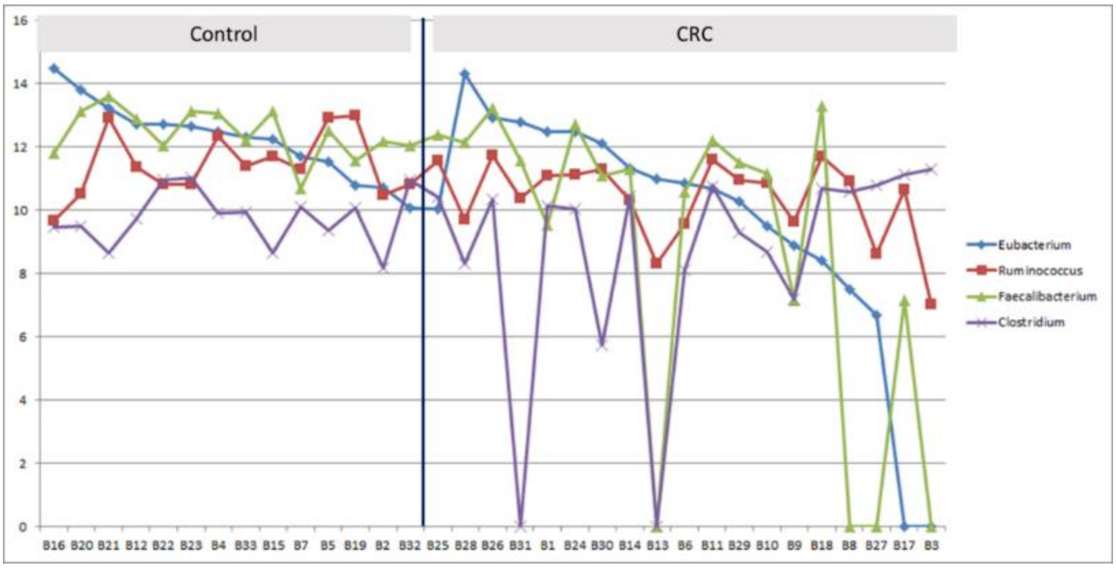

Four out of 20 genera that are more abundant are illustrated in a mutualistic pattern within the gut microbiota in LS CRC patients compared to their healthy 1st degree relatives. The X-axis designates sample ID and Y-axis shows the log mean fecal metagenomic abundances in samples. Each line represents an abundant genus.

Table S3. Curated species in LS CRC patients versus 1st-degree relatives

Id	baseMean	FoldChange	log2FoldChange	pvalue_adjusted
Butyrivibrio fibrisolvens [1634]	13.68	5.283897e-03	-7.564	0.01770
Erysipelotrichaceae bacterium 5_2_54FAA [1279]	26.26	4.001530e-03	-7.965	0.04976
Escherichia coli [390]	922.57	2.481637e+02	7.955	0.01127
Thioalkalimicrobium cyclicum [199]	33.89	2.841440e+02	8.15	0.01770

Table S4 Proportion of various Enterotypes in LS CRC patients versus 1st-degree relatives

	1st-degree relatives	CRC	*p*-value
ET_B (G1)	70%	26%	*p*<0.01
ET_P (G2)	20%	58%	*p*<0.01
ET_F (G3)	10%	16%	*p*<0.01

The separation of subjects as assigned by their status (Lynch CRC vs First-degree relatives) differed with respect to Enterotypes (Table S1): the majority of individuals with normal colonoscopy result were classified under ET_B, while CRC patients were primarily classified under ET_P and ET_F; more than 1/3 of LS CRCs were classified under ET_F.

Figure S4. Positive correlation between genera in the gut microbiota in LS families including 19 CRC and 15 first-degree relatives

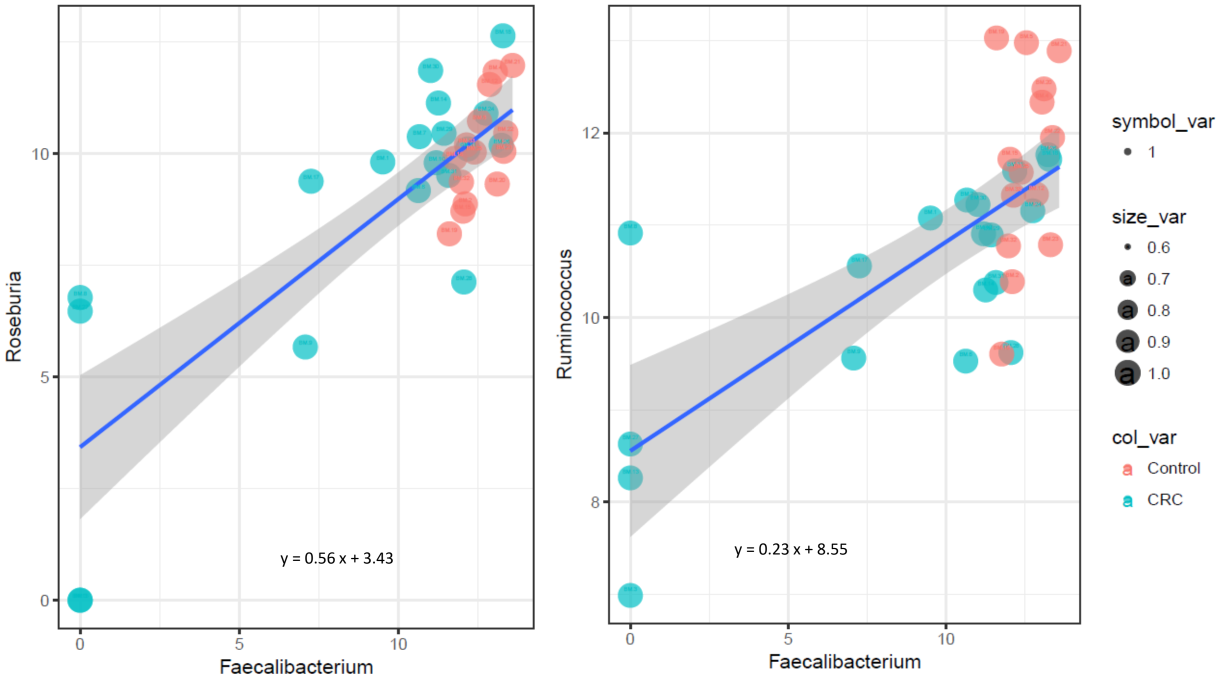

Id	Estmate	SD	t Value	p value
Faecalibacterium	0.55	0.07	7.8	0.00000007

Id	Estmate	SD	t Value	p value
Faecalibacterium	0.22	0.04	5.51	0.000004

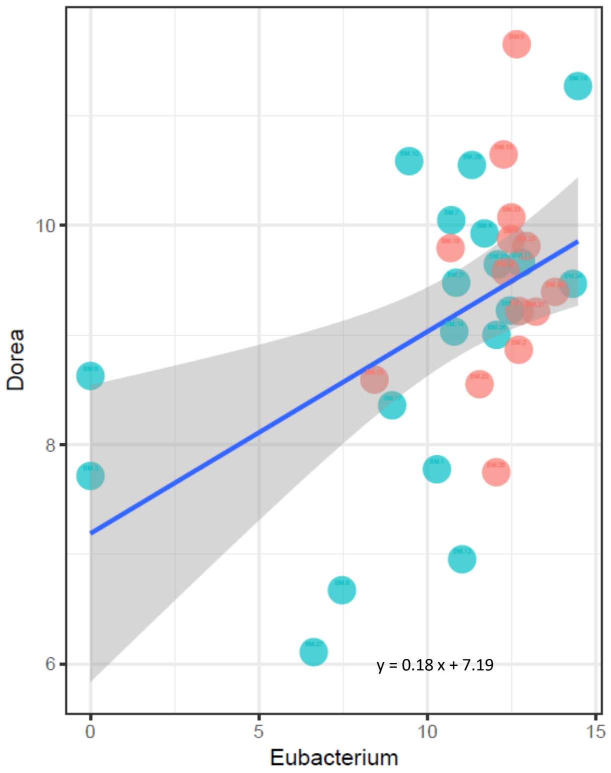

Id	Estmate	SD	t Value	p value
Eubacterium	0.18	0.05	3.14	0.0036

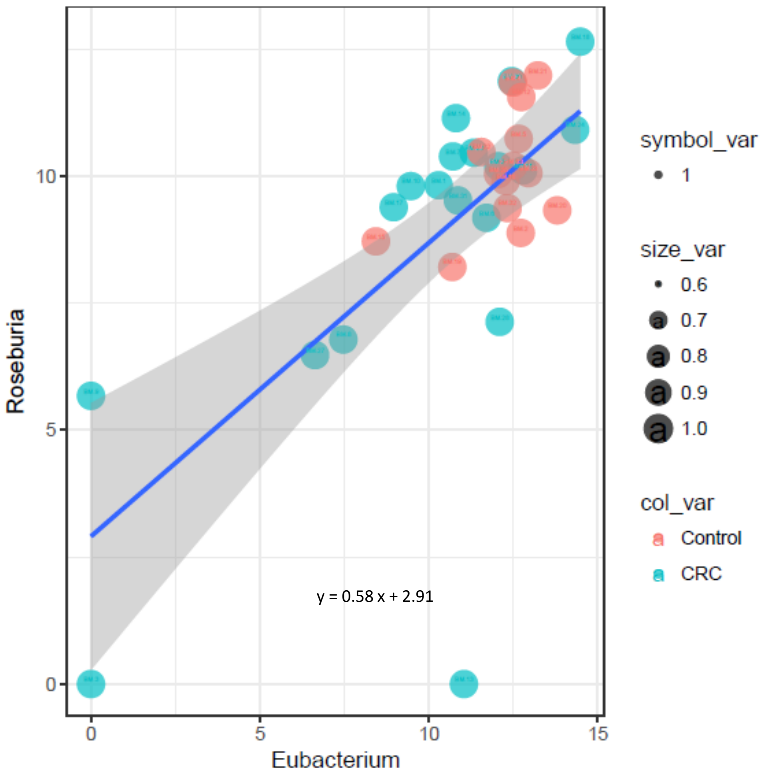

Id	Estmate	SD	t Value	p value
Eubacterium	0.57	0.11	5.08	0.000016

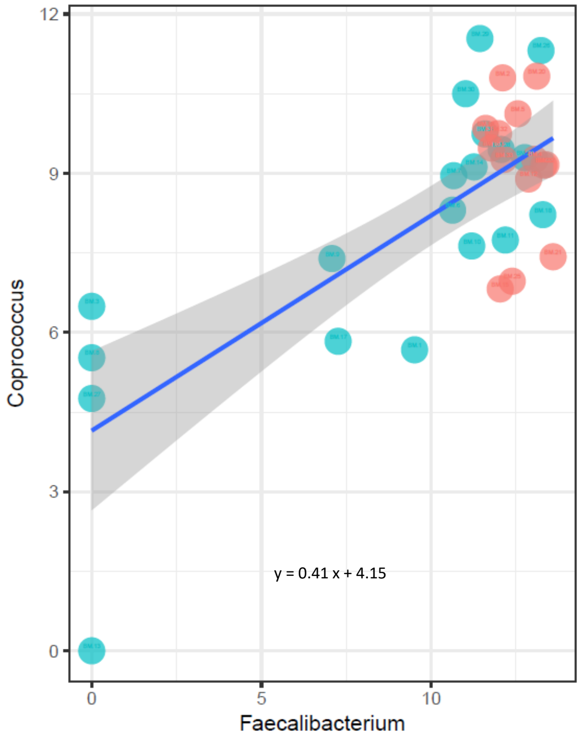

Id	Estmate	SD	t Value	p value
Faecalibacterium	0.40	0.06	6,14	0.00000008

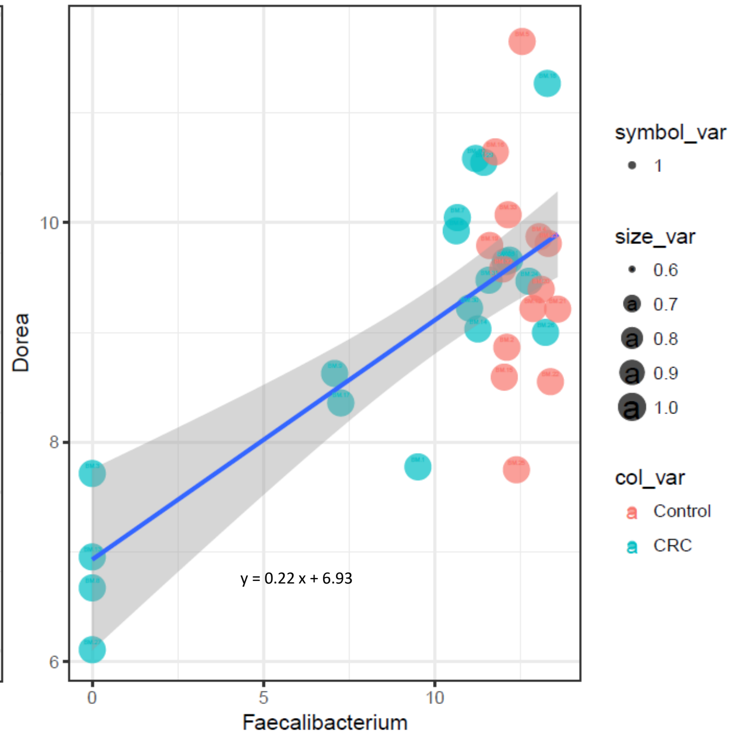

Id	Estmate	SD	t Value	p value
Faecalibacterium	0.22	0.03	5.99	0.0000012

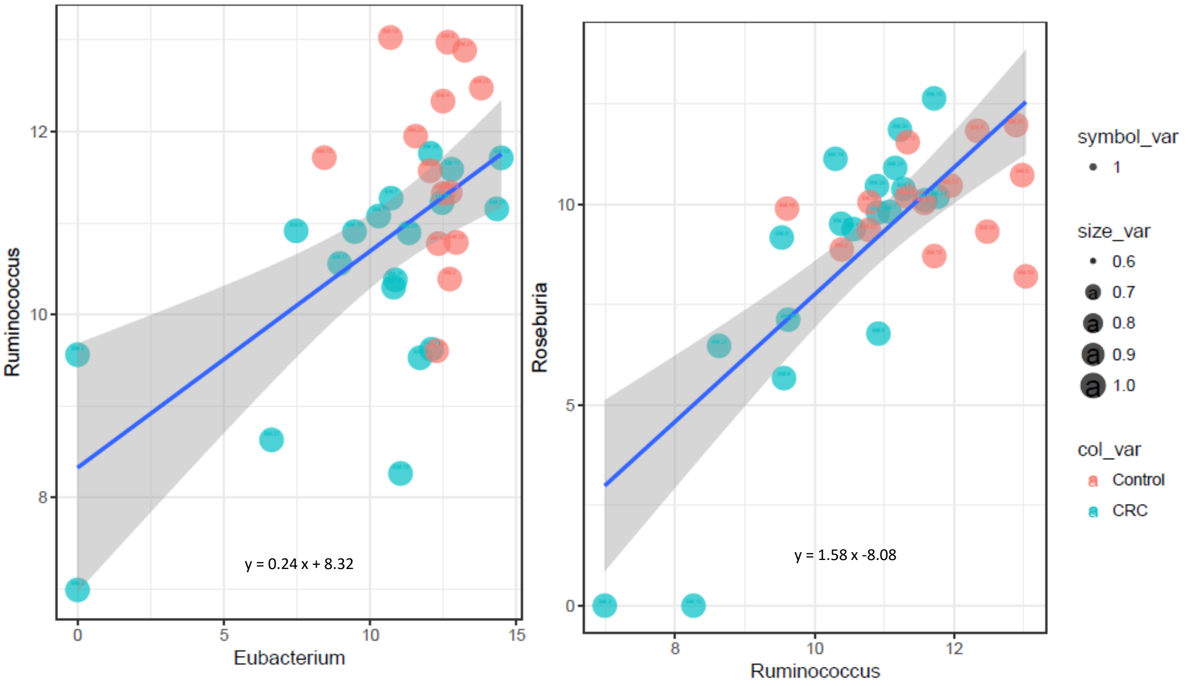

Id	Estmate	SD	t Value	p value
Eubacterium	0.27	0.05	4.02	0.00033

Id	Estmate	SD	t Value	p value
Eubacterium	1.56	0.25	6.25	0.00000006

Co-linked bacterial genera were identified among various symbiotics including butyrate-producer bacteria such as Ruminococcus, Roseburia, Eubacteria, Dorea and Faecalibacterium. The values are derived from the whole metagenomics analysis, using the Shaman webserver for visualization (see methods): CRC patients and first-degree relatives are indicated by blue and red circles, respectively. Blue lines illustrate correlations estimated using the Shaman webserver (Pearson correlation based on log-abundances of genera)

Figure S5. Heatmap illustration of main differential bacteria between LS CRC patients and first-degree relatives

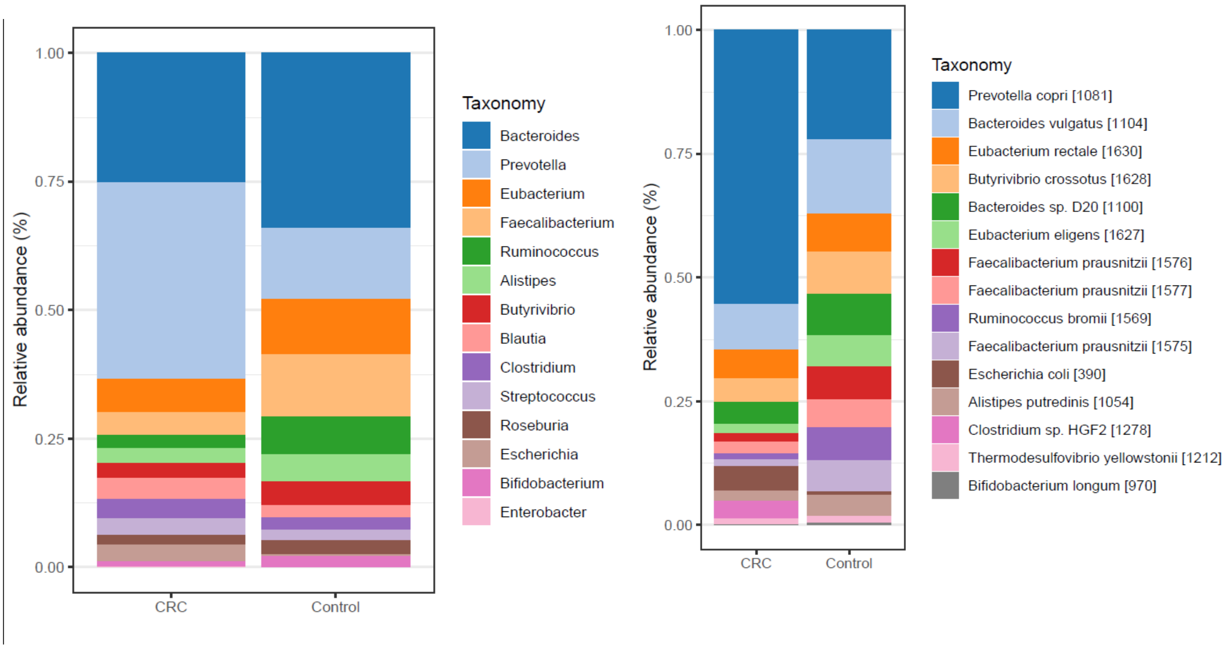

A. *Comparison regarding genera*

B. *Comparison regarding species*

Metagenomic analysis was performed on fresh stool samples from N=34 LS individuals (19 CRC cases; 15 first-degree relatives) within 17 different families

Table S5. Table including 4 LS families declaring to have exclusively national diet habits (C1: Chinese; C2: Lebanese; C3: French; C4: Portuguese)

	C2_vs_C3	C1_vs_C3	C1_vs_C4	C3_vs_C4
1	Akkermansia muciniphila [1008]	Clostridium sp. HGF2 [1278]	Akkermansia muciniphila [1008]	Bacteroides sp. 2_2_4 [1094]
2	Bacteroides caccae [1096]	Enterococcus faecalis [1363]		Bacteroides sp. D22 [1093]
3	Bacteroides clarus [1099]	Eubacterium siraeum [1564]		Streptococcus parasanguinis [1421]
4	Bacteroides finegoldii [1095]			
5	Bacteroides ovatus [1094]			
6	Bacteroides sp. 1_1_14 [1092]			
7	Bacteroides sp. 2_2_4 [1094]			
8	Bacteroides sp. 20_3 [1088]			
9	Bacteroides sp. D2 [1094]			
10	Bacteroides sp. D22 [1093]			
11	Bacteroides stercoris [1098]			
12	Bacteroides thetaiotaomicron [1092]			
13	Bacteroides xylanisolvens [1093]			
14	Haemophilus parasuis [308]			
15	Mitsuokella multacida [1653]			
16	Parabacteroides sp. D13 [1088]			
17	Prevotella bergensis [1066]			
18	Prevotella copri [1081]			
19	Prevotella melaninogenica [1074]			
20	Prevotella sp. Oral taxon 472 [1063]			
21	Streptococcus infantis [1417]			
22	Streptococcus parasanguinis [1421]			
23	Streptococcus salivarius [1377]			
24	Streptococcus vestibularis [1376]			
25	Veillonella dispar [1262]			

C1= Chine ; C2= Liban ; C3=France ; C4=Portugal

	CRC_vs_ first degree relatives	C3_vs_C1	C3_vs_C2	C3_vs_C4	C3_vs_C5	C3_vs_C7	C3_vs_C8	C3_vs_C11	C3_vs_C13
1	Clostridium methylpentosum [1572]	Butyrivibrio crossotus [1628]	Bacteroides caccae [1096]	Streptococcus thermophilus [1375]	Butyrivibrio crossotus [1628]	Butyrivibrio crossotus [1628]	Alistipes putredinis [1054]	Akkermansia muciniphila [1008]	Butyrivibrio crossotus [1628]
2	Enterococcus faecalis [1363]	Coprobacillus sp. 29_1 [1271]	Bacteroides sp. 3_1_33FAA [1104]		Eubacterium eligens [1627]			Butyrivibrio crossotus [1628]	Escherichia coli [390]
3	Escherichia coli [390]	Ruminococcus albus [1566]	Bacteroides sp. 3_1_40A [1104]		Ruminococcus albus [1565]			Ruminococcus albus [1565]	Faecalibacterium prausnitzii [1575]
4	Klebsiella oxytoca [401]	Ruminococcus flavefaciens [1567]	Bacteroides stercoris [1098]		Ruminococcus albus [1566]				Ruminococcus sp. 5_1_39BFAA [1620]
5	Ruminococcus albus [1565]				Ruminococcus flavefaciens [1567]				Ruminococcus torques [1610]
6	Ruminococcus albus [1566]								
7	Ruminococcus flavefaciens [1567]								
8	Shigella dysenteriae [390]								
9	Shigella flexneri [390]								
10	Veillonella atypica [1260]								

C1= Chine ; C2= Liban ; C3=France ; C4=Portugal

Table S6. Differential bacteria species in LS CRC patients and disease-free 1st degree relatives

Id	baseMean	Log2 FoldChange	pvalue_adjusted
Thermodesulfovibrio yellowstonii [1212]	320.6	1.436	1.467e-17
Ruminococcus sp. 5_1_39BFAA [1620]	2853.85	-1.729	0.000008
Ruminococcus obeum [1619]	598.08	-1.397	0.000180
Ruminococcus obeum [1618]	1284.05	-1.458	0.000271
Ruminococcus bromii [1569]	5135.72	-1.722	0.009961
Ruminococcus lactaris [1609]	666.97	-1.372	0.022029
Coprococcus comes [1616]	1040.92	-1.016	0.030145
Ruminococcus sp. SR1/5 [1621]	1546.82	-0.995	0.030153
Dorea longicatena [1605]	1629.25	-1.042	0.030767
Eubacterium hallii [1597]	1511.61	-1.237	0.030767
Ruminococcus sp. 5_1_39BFAA [1620]	2853.85	-1.729	0.000008

Figure S6. The correlation matrix average logarithm of CFU between *Bifidobacterium* and *Escherichia* in two groups of Yes and No

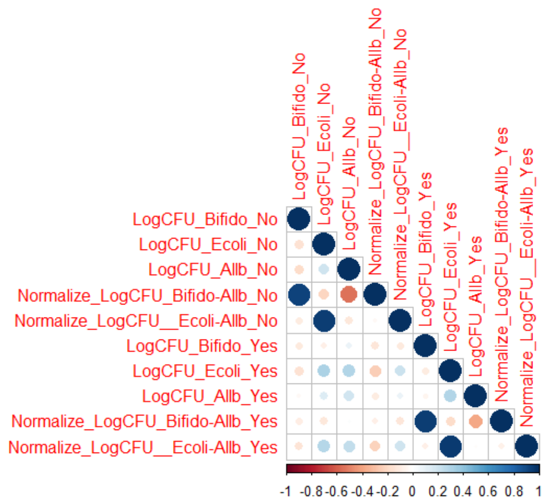

Positive correlations are displayed in blue, and negative correlations are in red. The intensity of the color and the size of the circles are proportional to the correlation coefficients.

Figure S7. Relative abundances (%) of phyla and genera in asymptomatic LS individuals' feces, according to colonoscopy.

A B

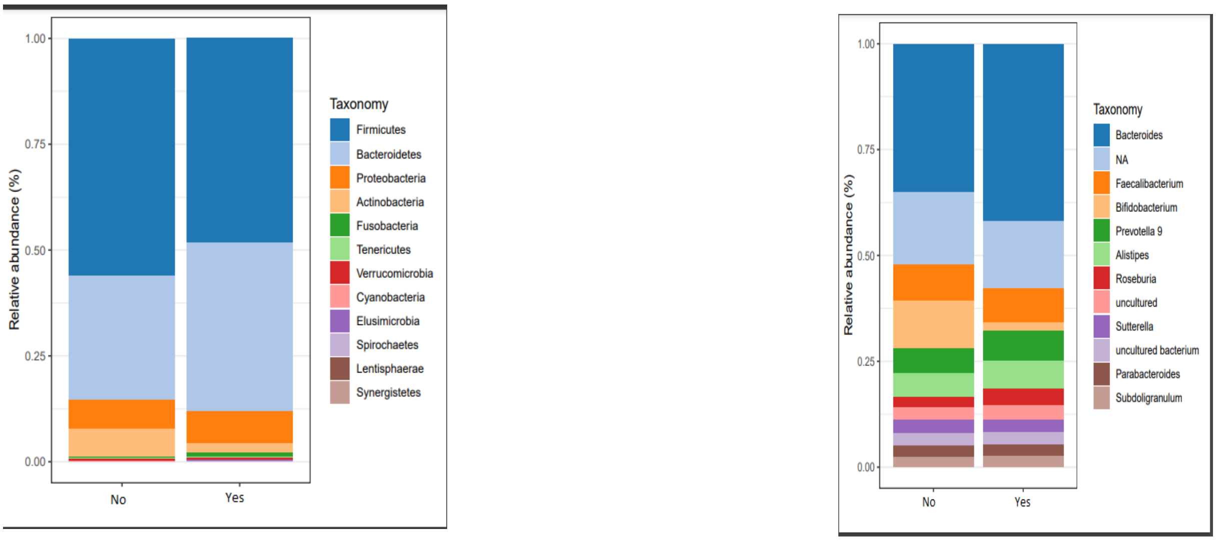

Barplot the phyla (A) and genera (B) abundances (% of total) in patients with (Yes) and those without (No) events (precancerous lesion) during colonoscopy

Table S7. Primers and probes used for qPCR.

Target organism	Primer & Probe	Sequence 5'-3'	Reference
All Bacteria	F_Bact1369<br>R_Prok1492<br>**P_ TM1389F**	CGGTGAATACGTTCCCGG<br>TACGGCTACCTTGTTACGACTT<br>**6FAM-CTT GTA CAC ACC GCC CGT C**	(Suzuki *et al.*,2000 [20])
Bifidobacterium	F_Bifid09c<br>R_Bifid06<br>**P_Bifid**	CGGGTGAGTAATGCGTGACC<br>TGATAGGACGCGACCCCA<br>**6FAM-CTC CTG GAA ACG GGT G**	(Furet et al, 2008 [18])
Escherichia coli	E.coli F<br>E.coli R	CAT GCC GCG TGT ATG AAG AA<br>CGG GTA ACG TCA ATG AGC AAA	(Huijsdens et al, 2002 [19])

The probe sequences are in bold characters.
